# Causal relationship between circulating immune cells and inflammatory bowel disease: A Mendelian randomization analysis

**DOI:** 10.1097/MD.0000000000039056

**Published:** 2024-07-26

**Authors:** Shan Li, Dujuan Mao, Quanshui Hao, Lijuan You, Xiufang Li, Yaohua Wu, Lai Wei, Heng Du

**Affiliations:** a Department of Anesthesiology, Huanggang Central Hospital Affiliated to Yangtze University, Huanggang, Hubei, China; b Anesthesiology Center, Hunan Provincial People’s Hospital (The First Affiliated Hospital of Hunan Normal University), Changsha, Hunan, China; c Department of Gastrointestinal Surgery, Huanggang Central Hospital Affiliated to Yangtze University, Huanggang, Hubei, China.

**Keywords:** causal association, genetic, immune cells, inflammatory bowel disease, Mendelian randomization

## Abstract

Inflammatory bowel disease (IBD) is an immune-mediated inflammation of the gastrointestinal tract that includes Crohn disease and ulcerative colitis (UC). Although IBD is associated with elevated levels of innate and adaptive immunity, the relationship between circulating immune cells and IBD remains largely unknown. Therefore, we conducted a bidirectional 2-sample Mendelian randomization (MR) study to determine their causal relationship. Genome-wide association study summary statistics were extracted from publicly available databases regarding immune cell phenotypes and IBD traits (including IBD, Crohn disease, and UC). MR analysis was conducted using 5 MR methods, with inverse-variance-weighted (IVW) as the primary analysis method. False discovery rate correction (FDR) was used to reduce the likelihood of type 1 errors. We also conducted MR-Egger-intercept tests to evaluate horizontal pleiotropy. After FDR adjustment of the *P* values for the IVW method, the results indicated no causal relationship between immune cell phenotypes and IBD or UC, but 4 immune characteristics were causally associated with Crohn disease. The percentage of human leukocyte antigen DR+ CD4+ T cells in lymphocytes was positively associated with the development of Crohn disease (odd ratio [OR], 1.13; 95% confidence interval [CI], 1.07–1.21; *P* < .001; *P*_FDR_ = 0.019), whereas the percentage of IgD− CD27− B cells in lymphocytes (OR, 0.85; 95% CI, 0.79–0.92; *P* < .001; *P*_FDR_ = 0.014), CD28 on CD39+ secreting CD4 regulatory T cells (OR, 0.92; 95% CI, 0.89–0.96; *P* < .001; *P*_FDR_ = 0.019), and the percentage of naïve CD4+ T cells in all CD4+ T cells (OR, 0.90; 95% CI, 0.85–0.95; *P* < .001; *P*_FDR_ = 0.027) were negatively related to the risk of Crohn disease. MR analysis of the above 4 immune cell phenotypes revealed no horizontal pleiotropy. In the reverse MR analysis, Crohn disease was not causally associated with any of these immune cell phenotypes. The findings provide insight into the relationship between immune cells and IBD pathogenesis, and may serve as a basis for developing novel immunotherapies.

## 1. Introduction

Inflammatory bowel disease (IBD) is an immune-mediated inflammation of the gastrointestinal tract that includes Crohn disease and ulcerative colitis (UC). It is estimated that there are 6 to 8 million cases of IBD worldwide.^[1]^ The inflammation associated with IBD is progressive and destructive, which can lead to complications such as fibrosis, stenosis, or cancer.^[2]^ Hence, it is imperative to investigate the underlying mechanisms of IBD and develop effective anti-inflammatory therapies.

IBD is characterized by heightened levels of innate and adaptive immunity in the gut in association with dysbiosis of the microbiota and disruption of the intestinal barrier.^[3]^ When microorganisms or damaged tissue provide signals to innate immune cells, these cells produce inflammatory cytokines and factors that stimulate the T and B cells of the adaptive immune system.^[3,4]^ There is an accumulation of inflammatory T cells and their pro-inflammatory-associated cytokines in the intestinal tissue of those with IBD.^[2]^ It has been established that immune cells play a critical role in the pathogenesis of IBD.^[2,3]^ However, immune cells can be divided into a number of subpopulations according to the presence of different immunological markers, and these subpopulations may function differently. Until now, the relationship between immune cell subpopulations and IBD remains largely unknown.

The Mendelian randomization (MR) method is a newly developed genetic epidemiological technique based on Mendel laws of genetic inheritance.^[5]^ In MR, genetic variations are utilized as instrumental variants, which are assigned by chance at conception, prior to the onset of the disease.^[6]^ MR can therefore identify causal relationships independent of confounding factors and avoid reverse causality.^[7]^ The use of MR analysis has increased in recent years for identifying potential IBD risk factors.^[8–11]^ A number of MR studies have also provided evidence linking inflammatory biomarkers to IBD, such as interleukin (IL)-17,^[12]^ IL-18,^[13,14]^ C-X–C motif chemokine 9,^[15]^ and C–C motif chemokine 23.^[13]^ Despite this, the relationship between circulating immune cells and the risk of IBD has not been thoroughly examined. Thus, we conducted a bidirectional 2-sample MR analysis using genome-wide association study (GWAS) data to determine the causal relationship between immune cell traits and IBD.

## 2. Methods

### 2.1. Study design

**Figure 1.**
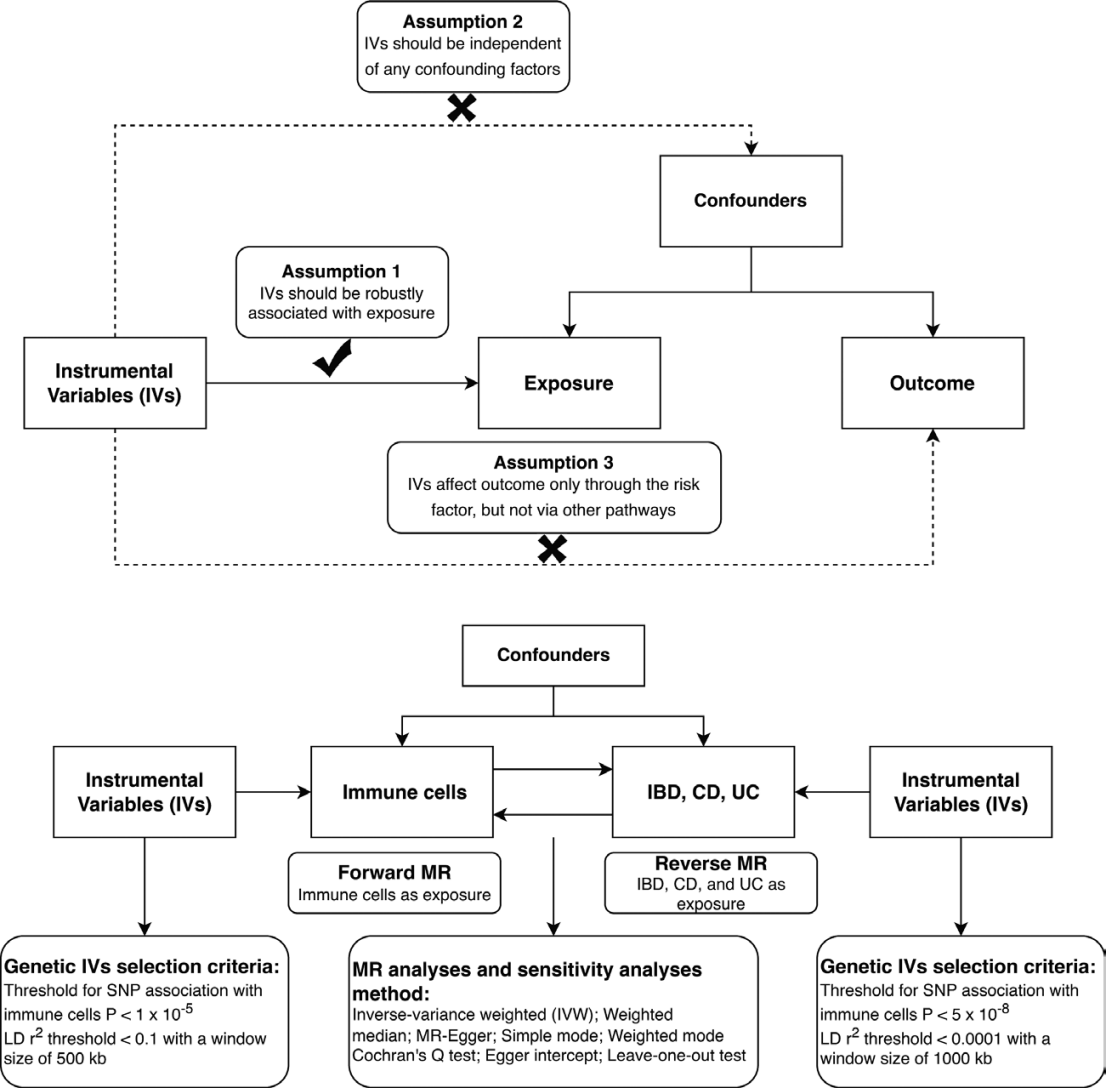
Three key assumptions of the MR study and the design of our study design. CD = Crohn disease, IBD = inflammatory bowel disease, IV = instrumental variant, MR = Mendelian analysis, SNP = single nucleotide polymorphism, UC = ulcerative colitis.

This is a bidirectional 2-sample MR study. A forward MR analysis considered immune cell traits as exposures, and IBD traits, such as IBD, Crohn disease, and UC, as outcomes, while a reverse MR analysis considered IBD traits as exposures, and immune cell traits as outcomes. Single nucleotide polymorphisms (SNPs) are used as instrumental variants (IVs) in MR, and valid IVs must meet the following 3 assumptions^[16–18]^: the relevance assumption requires IVs to be robustly associated with exposure; the independence assumption demands that IVs be independent of any confounding factors; the exclusion limitation assumption states that IVs only affect outcomes through the risk factor, not other pathways. Our study design is illustrated in Figure 1.

### 2.2. Data sources

GWAS-summary statistics for each immune trait are obtained through the GWAS Catalog (accession numbers GCST90001391 to GCST90002121).^[19]^ We analyzed a total of 731 immune cell phenotypes, which included absolute counts (n = 118), relative counts (n = 192), fluorescence intensities (n = 389), and morphological parameters (n = 32). In the GWAS on immune traits, 3757 European individuals were analyzed using 20,143,392 SNPs and the associations were examined after adjusting for covariates such as sex, age, and age^2^.^[20]^ GWAS-summary statistics on IBD, Crohn disease, and UC are available through the GWAS catalog (Accession Nos. GCST004131 to GCST004133). There are 25,042 cases and 34,915 controls for IBD, 12,194 cases and 28,072 controls for Crohn disease, and 12,366 cases and 33,609 controls for UC.^[21]^ The GWAS statistics were all derived from European individuals. Ethical approval for this project was not required as the data analyzed were publicly available, and each source of data had already obtained ethical approval from their respective institutions.

### 2.3. Selection of IVs

In accordance with previous studies,^[22,23]^ we extracted significant SNPs at a significance level of 1 × 10^−5^ for each immune trait. With the PLINK clumping procedure, we identified independent SNPs based on linkage imbalance (LD) *r*^2^ threshold < 0.1 with a window size of 500 kb,^[22]^ where LD *r*^2^ was calculated based on the 1000 Genomes Project reference panel.^[24]^ For GWAS data of IBD traits, we set a significance level of 5 × 10^−8^, and a LD *r*^2^ < 0.0001 with a window size of 1000 kb. An *F* statistic was calculated for each SNP to evaluate its strength. To avoid weak instrumental bias, only SNPs with *F* > 10 were included in the analysis as IVs.^[25]^ Additionally, we used the Phenoscanner V2 to eliminate SNPs that were directly associated with confounders and outcomes.^[26,27]^

### 2.4. Statistical analysis

Five MR analyses were conducted to examine causal relationships between various immune cell phenotypes and IBD traits: inverse-variance weighting (IVW), MR-Egger, weighted median, simple mode, and weighted mode, with IVW (random effects) being the primary analysis.^[28]^ To minimize the likelihood of type 1 errors in this study, we used the false discovery rate (FDR) correction, and a *P*_FDR_ < 0.05 was considered significant. Cochran *Q* statistic was employed to assess heterogeneity among selected IVs, and a *P* value < .05 indicates the existence of heterogeneity.^[28]^ We evaluated horizontal pleiotropy using the MR-Egger-intercept test. If the MR-Egger-intercept was significantly (*P* < .05), it suggested that association results may be influenced by horizontal pleiotropic effects of other traits.^[29]^ In order to visualize our results, forest plots, heatmaps, and scatter plots were generated. The leave-one-out sensitivity test was also performed to assess the influence of a single SNP on the causal estimates. Statistical analyses were conducted using R version 4.3.1 (http://www.Rproject.org).

## 3. Results

### 3.1. The causal effects between immune cell phenotypes and IBD

**Figure 2.**
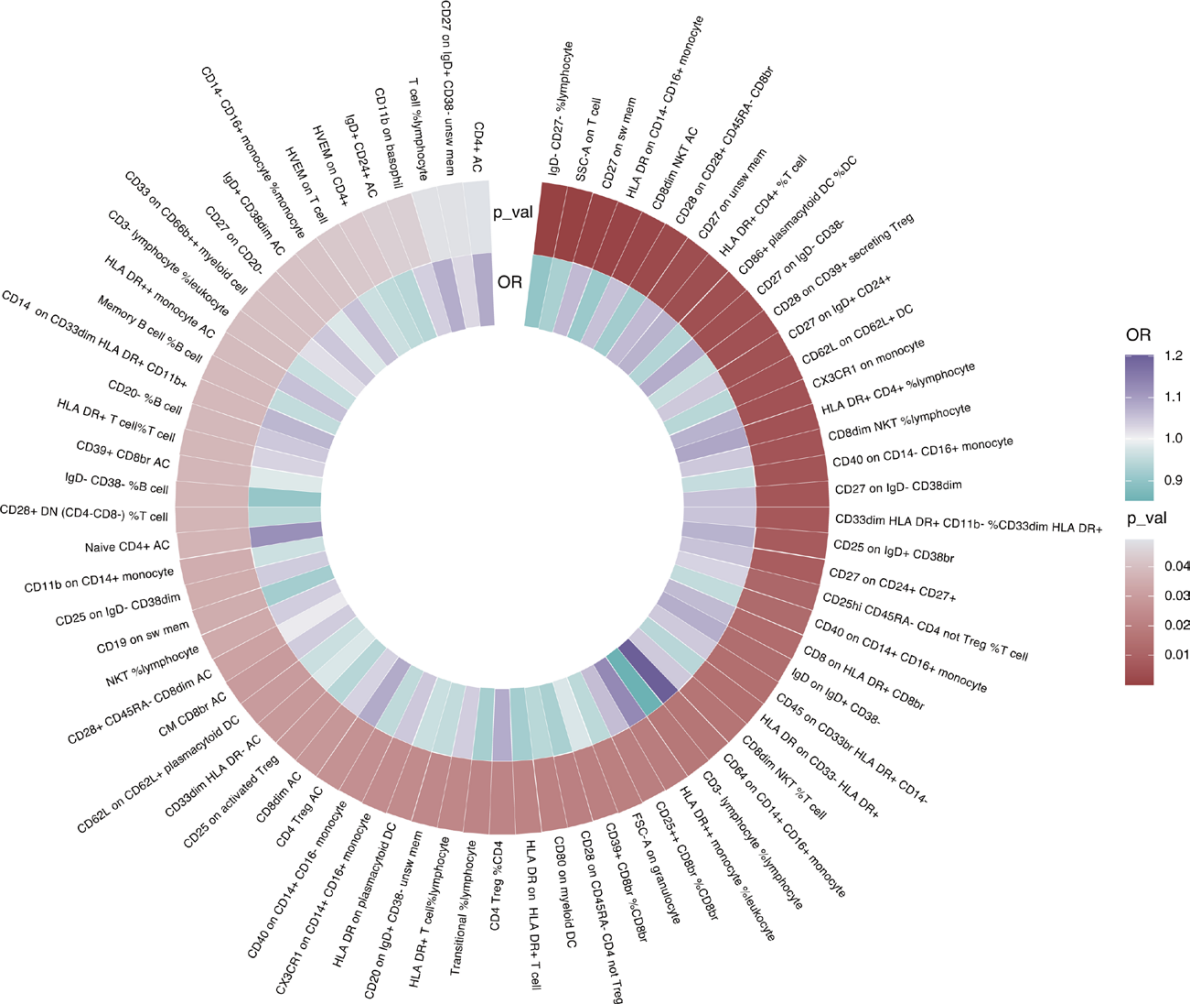
Heatmap for the causal effect of circulating immune cells on inflammatory bowel disease using the inverse-variance weighting (IVW) method. The outer circle represents *P* values, whereas the inner circle represents odds ratios (ORs).

We first examined the causal effects of immune cell phenotypes on IBD, and the IVW method was used as the primary analysis method. A total of 76 immune cell phenotypes were found to be associated with IBD (*P* < .05) (Fig. 2). However, following FDR adjustment, none of them was found to be causally associated with IBD. MR analysis using 4 other methods (MR-Egger, weighted median, simple mode, and weighted mode) is presented in Supplementary Table S1, Supplemental Digital Content, http://links.lww.com/MD/N263. Furthermore, we determined the horizontal pleiotropy and heterogeneity of each immune cell phenotype–IBD pairing, and the results are shown in Supplementary Table S2, Supplemental Digital Content, http://links.lww.com/MD/N263.

The causal effects of IBD on immune cell phenotypes were also examined. The FDR adjustment of *P* values for the IVW method revealed that IBD was not causally associated with any of the immune cell phenotypes (*P*_FDR_ > 0.05). In Supplementary Tables S3 and S4, Supplemental Digital Content, http://links.lww.com/MD/N263, we present the MR results, as well as the heterogeneity and horizontal pleiotropy results.

### 3.2. The causal effects between immune cell phenotypes and Crohn disease

**Figure 3.**
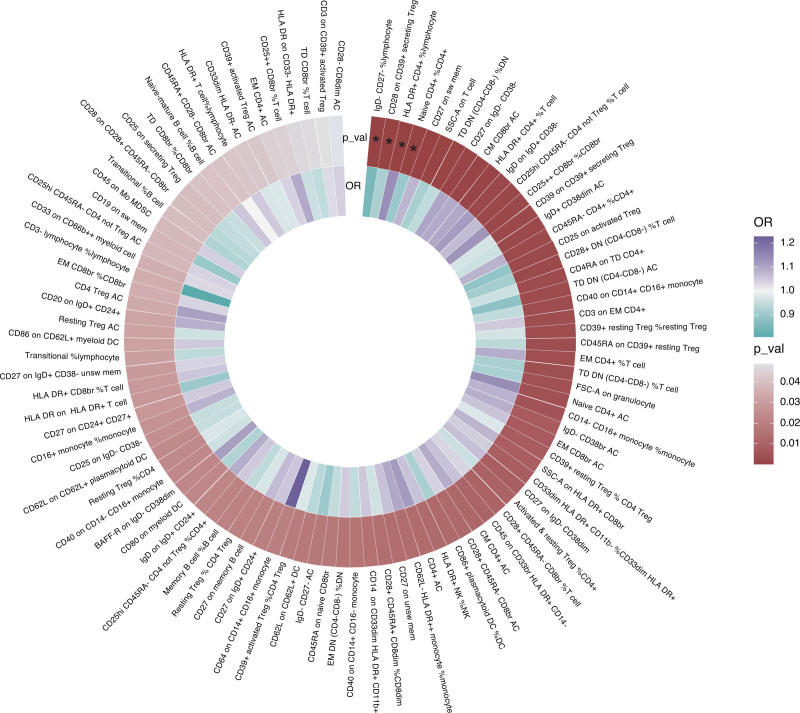
Heatmap for the causal effect of circulating immune cells on Crohn disease using the inverse-variance weighting (IVW) method. The outer circle represents *P* values, whereas the inner circle represents odds ratios (ORs). * indicates the false discovery rate corrected *P* values < .05.

**Figure 4.**
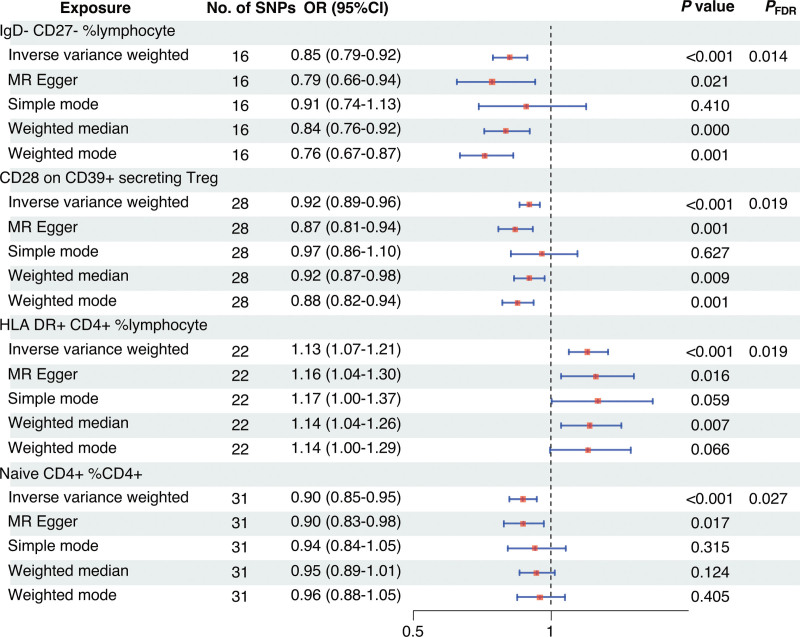
Forest plot for the causal effect of circulating immune cells on Crohn disease. CI = confidence interval, FDR = false discovery rate, MR = Mendelian randomization, OR = odds ratio, SNPs = single nucleotide polymorphisms.

**Figure 5.**
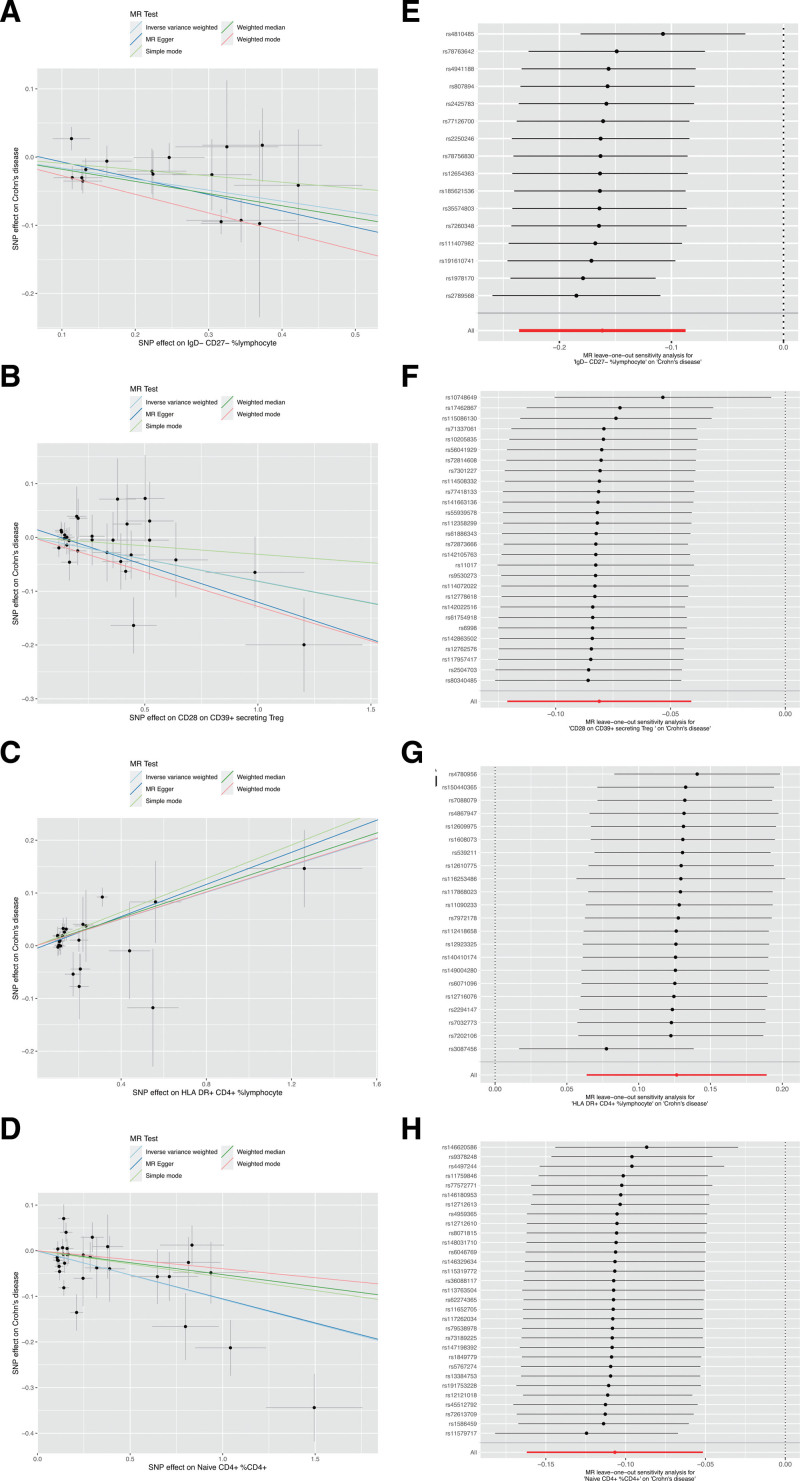
Scatter plots (A–D) and leave-one-out forest plots (E–H) for the causal effect of circulating immune cells on Crohn disease. MR = Mendelian randomization, SNP = single nucleotide polymorphism.

First, we examined the causal effects of immune cell phenotypes on Crohn disease. Based on the MR analysis using the IVW method, 97 immune cell phenotypes were associated with Crohn disease (*P* < .05) (Fig. 3). A causal association was determined between 4 immune characteristics and Crohn disease after adjustment for FDR (*P*_FDR_ < 0.05), namely: the percentage of IgD− CD27− B cells in lymphocytes, CD28 on CD39+ secreting CD4 regulatory T (Treg) cells, the percentage of human leukocyte antigen (HLA) DR+ CD4+ T cells in lymphocytes, and the percentage of naïve CD4+ T cells in all CD4+ T cells. As shown in Figure 4, the percentage of HLA-DR+ CD4+ T cells in lymphocytes was positively related to the development of Crohn disease, with an odds ratio (OR) of 1.13 (95% confidence interval [CI], 1.07–1.21; *P* < .001; *P*_FDR_ = 0.019); the percentage of IgD− CD27− B cells in lymphocytes (OR, 0.85; 95% CI, 0.79–0.92; *P* < .001; *P*_FDR_ = 0.014), CD28 on CD39+ secreting CD4 Treg cells (OR, 0.92; 95% CI, 0.89–0.96; *P* < .001; *P*_FDR_ = 0.019), and the percentage of naïve CD4+ T cells in all CD4+ T cells (OR, 0.90; 95% CI, 0.85–0.95; *P* < .001; *P*_FDR_ = 0.027) were negatively associated with the development of Crohn disease. Other 4 MR methods showed similar results. Figure 5A–D illustrates the scatter plots for the MR analysis of these immune cell phenotypes. While conducting MR analysis on the above 4 immune cell phenotypes, significant heterogeneity (Cochran *Q* statistic, *P* < .05) was observed when analyzing the percentage of naïve CD4+ T cells in all CD4+ T cells (Table 1). Based on the MR-Egger-intercept analysis, there was no significant horizontal pleiotropy (*P* > .05) for each immune cell phenotype (Table 1), suggesting that the SNPs did not influence the outcome via factors unrelated to the exposure factors. Moreover, the leave-one-out sensitivity test showed that the overall causal estimates were not affected by individual SNPs (Fig. 5E–H).

**Table 1:** Heterogeneity and horizontal pleiotropy results for the Mendelian randomization analysis of immune cell phenotypes on Crohn disease.

Exposure	*P* value (Q-Egger)	*P* value (Q-IVW)	Egger-intercept	*P* value (Egger-intercept)
IgD− CD27− %lymphocyte	.082	.083	0.017	.369
CD28 on CD39+ secreting Treg	.460	.595	0.018	.076
HLA-DR+ CD4+ %lymphocyte	.120	.102	−0.006	.597
Naive CD4+ %CD4+	<.001	<.001	−0.001	.957

HLA = human leukocyte antigen, IVW = inverse-variance weighted.

**Figure 6.**
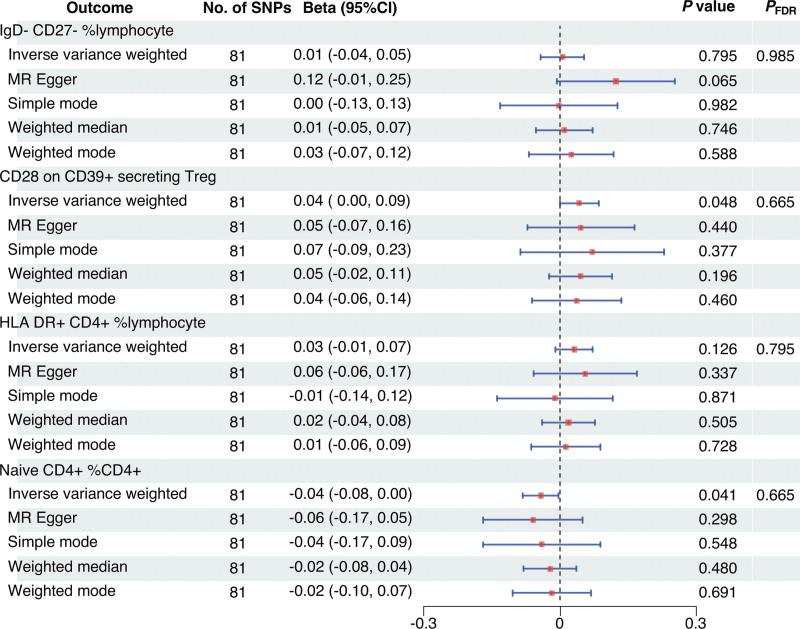
Forest plot for the causal effect of Crohn disease on circulating immune cells. CI = confidence interval, FDR = false discovery rate, MR = Mendelian randomization, SNPs = single nucleotide polymorphisms.

We then examined the causal effects of IBD on immune cell phenotypes. Based on FDR adjustment of *P* values for the IVW method, Crohn disease showed no causal association with any of the immune cell phenotypes (*P*_FDR_ > 0.05), including the 4 immune cell phenotypes described above (Fig. 6).

The MR results as well as heterogeneity and horizontal pleiotropy results for all immune cell phenotypes are presented in Supplementary Tables S5–S8, Supplemental Digital Content, http://links.lww.com/MD/N263.

### 3.3. The causal effects between immune cell phenotypes and UC

**Figure 7.**
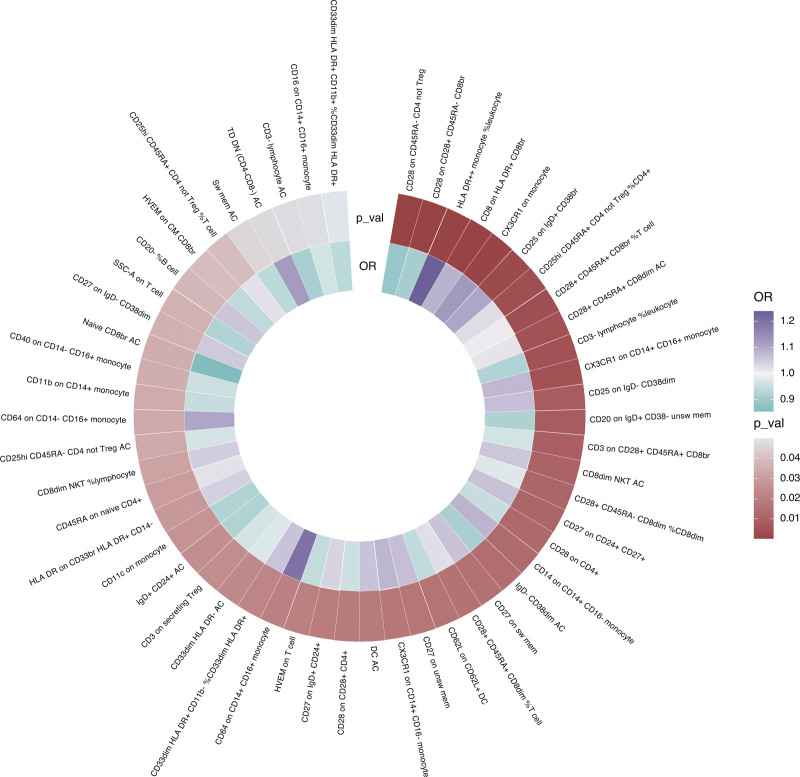
Heatmap for the causal effect of circulating immune cells on ulcerative colitis using the inverse-variance weighting (IVW) method. The outer circle represents *P* values, whereas the inner circle represents odds ratios (ORs).

First, we examined the causal effects of immune cell phenotypes on UC. According to the MR analysis using the IVW method, 53 immune cell phenotypes were associated with UC (*P* < .05) (Fig. 7). Nevertheless, after adjusting for FDR, none of them showed a causal association with UC. Supplementary Table S9, Supplemental Digital Content, http://links.lww.com/MD/N263 presents the results of MR analysis using the other 4 methods (MR-Egger, weighted median, simple mode, and weighted mode). Supplementary Table S10, Supplemental Digital Content, http://links.lww.com/MD/N263 shows the results of heterogeneity and horizontal pleiotropy analyses for each immune cell phenotype-UC pair.

The causal effects of UC on immune cell phenotypes were then examined. After FDR adjustment of the P values for the IVW method, UC did not show any causal association with any of the immune cell phenotypes (*P*_FDR_ > 0.05). Supplementary Tables S11 and S12, Supplemental Digital Content, http://links.lww.com/MD/N263 show the results of the MR analysis as well as the heterogeneity and horizontal pleiotropy analyses.

## 4. Discussion

In the present study, we used MR to investigate the causal relationship between immune cell traits and IBD, Crohn disease, as well as UC. According to our study, the percentage of HLA-DR+ CD4+ T cells in lymphocytes was positively associated with the development of Crohn disease, whereas IgD− CD27− B cells in lymphocytes, CD28 on CD39+ secreting CD4 Treg cells, and percentage of naïve CD4+ T cells were negatively related to the development of Crohn disease.

In our study, we found that IgD− CD27− B cell counts were negatively associated with the risk of Crohn disease. IgD− CD27− B cells are a rare subset of B cells known as double-negative B cells. They constitute approximately 5% of all peripheral B cells.^[30]^ Although IgD− CD27− B cells are relatively rare in normal peripheral blood, they play an important role in a number of diseases, including systemic lupus erythematosus,^[31]^ coronavirus disease 2019,^[32]^ and malaria.^[33]^ Their functions include defending the host against pathogens as well as initiating autoimmune responses.^[34]^ This double-negative subset has been described as responsive to anti-inflammatory treatment,^[35,36]^ although the cause of the increase is not known. However, interestingly, a recent study showed that CD27− IgD− B cells in blood were reduced in patients with IBD,^[37]^ but the authors also reported that CD27− IgD− B cells in gut-associated lymphoid tissue were raised. Therefore, CD27− IgD− B cells may be recruited from the circulation into the gut, where they may contribute to the intestinal inflammatory milieu associated with IBD. Our study showed consistent findings with this study, which found that peripheral blood IgD− CD27− B cell counts are negatively correlated with the risk of Crohn disease. These findings, however, are preliminary, and more research is necessary to determine the role and underlying mechanisms of IgD− CD27− B cells in the development of IBD.

We found that CD28 expression on CD39+ secreting CD4 Treg cells in the peripheral blood was negatively associated with the risk of Crohn disease. Treg cells are a subset of CD4 T cells with anti-inflammatory properties,^[38]^ and are thought to be involved in the pathophysiology of IBD.^[39]^ It has been demonstrated in a previous study that there is an increase in CD4+ Treg cells in the inflamed tissues of patients with IBD.^[40]^ The increase may be the result of a compensatory mechanism to control the exacerbated pro-inflammatory immune response.^[39,40]^ CD39 has primarily been described as a Treg marker, whose hydrolysis of extracellular adenosine triphosphate plays a crucial role in their immunosuppressive function.^[41]^ In our study, CD28 expression was causally associated with Crohn disease in these Treg cells. CD28 plays a vital role in promoting proliferation and the function of conventional T cells. However, CD28 may also function as a pro-inflammatory or anti-inflammatory role depending on the type of cell and the context in which it is expressed.^[42]^ CD28 is required for both thymic development and peripheral homeostasis of Treg cells.^[43]^ It has been demonstrated that CD28 prevents spontaneous autoimmunity by promoting the anti-inflammatory function of the Treg cells.^[44]^ CD28 plays such an important role in the anti-inflammatory effects of Treg cells that it is reasonable to assume that CD28 expression on Treg cells is inversely associated with the risk of Crohn disease.

Our study indicated that the proportion of HLA-DR+ CD4+ T cells in lymphocytes was positively related to the development of Crohn disease, while the proportion of naïve CD4+ T cells was negatively related. It has been increasingly recognized that CD4+ T cells play a crucial role in immune pathogenesis in the intestinal mucosa of individuals with IBD,^[39,45]^ and HLA-DR expression on CD4+ T cells is an indication of their activation. According to a recent study, CD4+ T cell activation in the periphery may also contribute to the pathogenesis of IBD,^[46]^ but CD4+ T cells were found to be increased in Crohn disease but not in UC. In addition, they found that the proportions of HLA-DR+ T cells were correlated in both CD4+ and CD8+ T cell populations in patients with IBD.^[46]^ In addition, as a member of class II major histocompatibility complex (MHC) proteins,^[47]^ studies indicate that HLA-DR is closely associated with Crohn disease, and that Crohn disease susceptibility may be associated with specific allotypes of the human MHC.^[48]^ Consequently, it may be concluded that an increase in HLA-DR+ CD4+ T cells contributes to the development of Crohn disease. CD4+ T cells are all differentiated from naïve CD4+ T cells. Upon stimulation, naïve CD4+ T cells can differentiate into various types of cells that have different functions.^[49]^ The increase in naïve CD4+ T cells indicates their nonactivated state. Thus, the high proportion of naïve CD4+ T cells was associated with reduced risk of Crohn disease, which is in accordance with our findings.

In the present study, we used large-scale GWAS data, examined the association between more than 700 immune cell traits and IBD traits and provided genetic evidence for the causal relationship between circulating immune cells and IBD, Crohn disease, as well as UC. A number of immune cell traits were found to be causally related to the risk of Crohn disease. These findings may provide new insights into the immune mechanism of IBD and promote further research into corresponding immunotherapies. However, our study has several limitations. The study was conducted on the basis of data from a European database. The results may not be generalizable to other ethnic groups. Second, although we observed a causal relationship between certain immune cells and Crohn disease, the mechanisms underlying this relationship are still poorly understood, so our findings should be interpreted with caution.

## 5. Conclusions

In conclusion, our study demonstrated a causal relationship between certain immune cell characteristics and the development of Crohn disease. These findings provide insight into the relationship between immune cells and the pathogenesis of Crohn disease, and may serve as a basis for the development of immunotherapies for the treatment of Crohn disease. However, further research is necessary to confirm these causal relationships and to investigate the mechanisms underlying them.

## Acknowledgments

We would like to thank all the investigators who made their GWAS data publicly available.

## Author contributions

**Conceptualization:** Shan Li.

**Methodology:** Shan Li, Dujuan Mao.

**Visualization:** Shan Li, Quanshui Hao.

**Writing – original draft:** Shan Li, Dujuan Mao.

**Validation:** Dujuan Mao, Lijuan You, Xiufang Li.

**Software:** Quanshui Hao, Lijuan You, Xiufang Li.

**Supervision:** Yaohua Wu, Lai Wei.

**Writing – review & editing:** Yaohua Wu, Lai Wei, Heng Du.

**Funding acquisition:** Lai Wei, Heng Du.

## Supplementary Material
